# Risk Factors for Recurrent Violent Injuries Among African Women in The Gambia

**DOI:** 10.5811/westjem.2022.4.54880

**Published:** 2022-07-05

**Authors:** Paul Bass, Wen-Yu Yu, Sy-Jou Chen, Edrisa Sanyang, Mau-Roung Lin

**Affiliations:** * University of The Gambia, School of Medicine & Allied Health Sciences, Department of Public & Environmental Health, Brikama, The Gambia; † Taipei Medical University, College of Public Health, Graduate Institute of Injury Prevention and Control, Taipei, Taiwan; ‡ Taipei Medical University Hospital, Department of Emergency Medicine, Taipei, Taiwan; § National Defense Medical Center, Tri-Service General Hospital, Department of Emergency Medicine, Taipei, Taiwan; ¶ Western Kentucky University, College of Health and Human Services, Department of Public Health, Bowling Green, Kentucky

## Abstract

**Introduction:**

Violence against women remains a major public health concern in African countries. We conducted a matched case-control study to identify risk factors for recurrent violent injuries among African women in The Gambia, a small West African country.

**Methods:**

During the 12-month study period, we recruited study participants from eight emergency departments in the metropolitan areas of the municipality of Kanifing and the West Coast region. We selected women aged ≥15 years who sought medical treatment for an injury due to physical violence at least twice over the study period. Two control groups were used: violence controls (VC), which included those who had experienced a single violence-related injury in the prior 12 months; and nonviolence controls (NVC), which included those who had experienced a nonviolent injury. Control patients were matched based on gender, health facility, injury date, and age (±2 years).

**Results:**

In total, 116 case patients and 232 control patients participated in the study. Results of the conditional logistic regression analyses of the VC and NVC control groups individually showed that women with recurrent violent injuries had a significantly higher likelihood of having a secondary education (odds ratio [OR]_VC_ 6.47; OR_NVC_ 4.22), coming from a polygamous family (OR_VC_ 3.81; OR_NVC_ 3.53), and had been raised by a single parent (OR_VC_ 5.25; OR_NVC_ 5.04). Furthermore, compared with the VC group, women with recurrent violent injuries had a significantly higher likelihood of living in a rented house (OR_VC_ 4.74), living with in-laws (OR_VC_ 5.98), and of having experienced childhood abuse (OR_VC_ 2.48). Compared with the NVC group, women with recurrent violent injuries had a significantly higher likelihood of living in an extended family compound (OR_VC_ 4.77), having more than two female siblings (OR_VC_ 4.07), and having been raised by a relative (OR_VC_ 3.52).

**Conclusion:**

We identified risk factors for recurrent injuries from physical violence among African women in The Gambia. Intervention strategies targeting these risk factors could be effective in preventing recurrent violence against African women.

## INTRODUCTION

Physical violence among women, which significantly affects their health and well-being, is a major public health concern, and it imposes a huge cost on society.^1^,^2^ Globally, one in three women experiences physical violence requiring emergency department (ED) treatment, and those with prior injury have a high risk of subsequent violent injury and death.^4^,^5^,^6^ Up to 60% of women in the Unites States treated in the ED due to physical violence injuries experience recurrent violence, and Black women are approximately three times more likely than White women to become victims of violence.^7^,^8^,^9^ In the United Kingdom, women account for 80% of ED trauma patients with recurrent physical violence.^10^,^11^

Studies conducted in North America and Europe have identified several risk factors – sociodemographics, lifestyle choices, and behavior – that are linked to recurrent violence against women,^5^,^8^,^12^–^19^ but risk factors for recurrent violence against African women have not been investigated. Furthermore, developed countries differ tremendously from African countries in terms of gender inequality, social cultures and behaviors, and economic aspects; hence, risk factors and their relevant interventions identified in developed countries may not be appropriately generalized to African countries. For example, the predominant sociocultural norms of patriarchy in African societies implicitly promote inequality between men and women and justify violence against women as well as create a social environment conducive to recurrent physical violence against women.^20^–^22^ To prevent the cycle of physical violence among African women, identifying risk factors for recurrent violence specifically in this population is necessary.

We conducted a matched case-control study to identify potential risk factors for recurrent injuries due to physical violence among African women in The Gambia, a small country in West Africa.

## METHODS

### Study Participants

During the 12-month period October 2016–September 2017, female patients aged ≥15 years who presented to the EDs of eight public healthcare facilities in the metropolitan areas of Kanifing Municipality and the West Coast region of The Gambia were selected to participate in this study: Serrekunda General Hospital; Brikama District Hospital; Faji Kunda (major health center); and Gunjur, Sukuta, Bakau, Banjul’nding, and Serrekunda (with smaller health centers). People living in these metropolitan areas account for about 60% of the country’s total population.^23^ We excluded private healthcare facilities because they do not offer 24-hour ED services to all patients. Furthermore, the study excluded patients who visited the ED for non-newly incurred injuries (eg, visited the ED a second time for the same violent injury), had difficulty communicating, or could not provide written informed consent.

Women aged ≥15 years who sought medical treatment in the EDs of the 15 healthcare facilities for injuries sustained as a result of physical violence at least twice over the prior 12 months were recruited to the case group. A violent injury was defined as any injury or physical pain that was intentionally inflicted by another person.^3^

We used two separate control groups of patients, one with violence-related and one with nonviolence-related injuries. For each case, two controls were selected, a violence control (VC) cohort that included women aged ≥15 years who had visited the same ED due to a violent injury only once in the prior 12 months, and a nonviolence control (NVC) cohort, which included women aged ≥15 years who visited the same ED due to a nonviolent injury, such as traffic collisions, falls, and sports. Once a case was identified and the patient recruited, two separate controls (VC and NVC controls) were identified and recruited from those who visited the same ED within the next 24 hours. In addition to gender, the two control groups were matched with the index case by the health facility, injury date, and age (±2 years). Matching the health facility and injury date was presumed to exclude potential confounding effects of geographical area and calendar time (weekday, weekend, and holiday). In total, 116 patients were recruited to the case, VC, and NVC groups each.

*Population Health Research Capsule*

What do we already know about this issue?

*Studies in the US and Europe have identified risk factors for recurrent violence against women, but few have been conducted regarding African women.*

What was the research question?

*Our goal was to identify risk factors for recurrent injuries due to physical violence among African women in The Gambia.*

What was the major finding of the study?

*Common risk factors for recurrent violent injuries in African women were having higher levels of education, living in a polygamous family, and being raised by a single parent.*

How does this improve population health?

*Specific intervention strategies targeting these risk factors could be effective in preventing violence against women in African countries.*

This research was reviewed and approved by the University of The Gambia Research and Publication Committee and The Gambia Government/Medical Research Council Joint Ethics Committee on Human Subjects’ Research. All participants provided written informed consent.

### Data Collection

Study variables of interest were obtained through personal interviews by emergency physicians and nurses. Once a case or a control was ascertained, a personal interview with a structured questionnaire at the ED collected relevant information immediately prior to the violent event with minimal memory lapses and recall errors. We conducted a four-hour training course, including the demonstration and practice of asking questions, probing, and recording responses and interview simulation, to ensure that the physicians and nurses understood the key areas of the structured questionnaire and the interpretation of key variables. To sustain data quality, each study site was regularly visited by two members of our research team (PB and ES) at two-week intervals during the 12-month study period to check for questionnaire completeness.

We collected information on sociodemographics and behavioral and social characteristics. Sociodemographic factors included the following: age; height; weight; gender, ethnicity; educational level; type of family origin: monogamous family (in which a husband lived with one spouse) or polygamous family (a husband living with multiple spouses); marital status; age at first marriage; employment status; household income level; number of households in the compound; numbers of male and female siblings; childhood upbringing; residential status (family house, rented house, or owned house); living with an in-law in the prior 12 months; and body mass index (BMI) (computed as weight in kilograms divided by height squared). Behavioral and social characteristics comprised cigarette smoking, alcohol consumption, illicit drug use in the prior week, witnessing parental violence, having been physically abused as a child, social support, and risk-taking behaviors.

We assessed social support using the Multidimensional Scale of Perceived Social Support (MSPSS).^24^ The 12-item MSPSS assesses an individual’s perception of support from family, friends, and significant others, with a seven-point rating scale for each item. Summative scores for each source range from 4 to 28, with a higher score indicating stronger social support. The MSPSS has excellent internal reliability (alpha coefficients of 0.91–0.94) and validity in a wide range of African settings.^24^, ^25^

Risk-taking behaviors were assessed using the revised domain-specific risk-taking scale (DOSPERT). The 30-item DOSPERT evaluates the likelihood that respondents might engage in behaviors from six domains (ethical, gambling, investing, health/safety, recreational, and social), with a seven-point rating scale for each item.^26^ A high score for each of the six domains indicates a high risk-taking level. The DOSPERT scale has been used in a wide spectrum of studies examining behavioral risk intentions among different age groups and has exhibited good reliability (alpha coefficients of 0.63–0.75) and validity in the African population.^27^

### Statistical Analysis

We compared sociodemographics and behavioral and social characteristics between case and control patients using Pearson’s chi-squared test for categorical variables and Student’s t-test for continuous variables. In addition, sociodemographics were compared between eligible case patients who did not participate and those who participated in the study.

A conditional logistic regression model was applied to investigate independent relationships of potential risk factors for recurrent violent injuries in which we computed adjusted odds ratios (OR) and their 95% confidence intervals (CI) after adjustment for potential confounding factors. In the initial multivariable analysis, variables with a *P*-value of <0.25 in the bivariable logistic analysis were included to minimize large type II errors in selection and bias inferences.^28^ We employed stepwise selection in the multivariable analysis, and retained variables with a final *P*-value of <0.05 in the final model. The likelihood ratio and Hosmer-Lemeshow goodness of fit tests were used to evaluate the appropriateness of the model. We performed all data analyses using Statistical Analysis Software version 9.4 (SAS Institute Inc, Cary, NC).

## RESULTS

Of 124 women identified to have a recurrent violent injury over the 12-month period, 116 (93.5%) agreed to participate in the study, of whom 96 (83%) had sought treatment more than twice at an ED for a violent injury in the prior 12 months. In addition, 116 patients each were recruited to the two control groups, namely the VC and NVC groups. Table 1 presents the distributions of sociodemographic characteristics of the case group and two control groups. Between the case and VC groups, we observed significant differences in education level, family origin type, living in an extended family compound, and childhood upbringing. Between the case and NVC groups, significant differences were observed in ethnicity, family origin type, educational level, living in an extended family compound, number of female siblings, and childhood upbringing. Between the case and two control groups, no significant differences were observed in age, marital status, age at first marriage, employment status, number of male siblings, and BMI. In addition, the case and VC groups were similar in terms of ethnicity and number of female siblings.

Table 2 presents the distributions of behavioral and social characteristics between the case and two control groups. The case and VC groups were similar in terms of perceived social support from family, friends, and significant others. Between the case and NVC groups, significant differences were observed in perceived social support from family members and friends, and no significant difference was detected in perceived social support from significant others. Furthermore, no significant differences were detected in risk-taking behaviors between the case and two control groups. Table 3 presents the distributions of family factors between the case and VC groups. Between the case and VC groups, a significant difference was detected in residential status, living with in-laws in the prior 12 months, witnessing parental violence, and having been physically abused as a child.

Table 4 presents the results of conditional logistic regression analyses obtained using two control groups (VC and NVC) individually for recurrent violent injuries among Gambian women. According to the VC group, participants who attained secondary education had a higher risk of recurrent violent injuries (OR 6.47; 95% CI 2.23–18.84) than those with primary or no formal education. Furthermore, participants belonging to a polygamous family had a higher risk of recurrent violent injuries (OR 3.81; 95% CI 1.42–10.26) than those belonging to a monogamous family. Participants raised by a single parent had a higher risk of recurrent violent injuries (OR 5.25; 95% CI 2.08–18.91) than those raised by both parents. Participants living in a rented house had a higher risk of recurrent violent injuries (OR 4.74; 95% CI 1.90–11.81) than those living in a family house. Participants living with in-laws in the prior 12 months (OR 5.98; 95% CI 2.14–16.74) or who had suffered physical abuse as a child (OR 2.48; 95% CI 1.01–6.10) had a higher risk of recurrent violent injuries than did their counterparts.

According to the NVC group, participants with secondary education (OR 4.22; 95% CI 2.67–10.68), from a polygamous family (OR 3.53; 95% CI 1.56–8.00), living in an extended family compound (OR 4.77; 95% CI 2.73–13.17), and having more than two female siblings (OR 4.07; 95% CI 2.81–9.17) had a higher risk of recurrent violent injuries than did their counterparts. Furthermore, participants raised by a single parent (OR 5.04; 95% CI 2.43–17.78) or a relative (OR 3.52; 95% CI 1.00–12.43) had a higher risk of recurrent violent injuries than those raised by both parents.

## DISCUSSION

Few studies have investigated potential risk factors for recurrent violent injuries among African women as a precursor to providing interventional strategies on violence prevention in the population. The use of two control groups allowed us to validate the result from one group and identify consistent risk factors for recurrent violent injuries. The results of this study indicate that African women have a significantly increased risk of recurrent injuries due to physical violence if they belonged to a polygamous family, attained secondary education, lived in an extended family compound, had more than two female siblings, were raised by a single parent or a relative, lived in a rented house, lived with in-laws in the prior 12 months, and had suffered physical abuse. Furthermore, significant differences were observed in sociodemographic and social characteristics between African women who had suffered recurrent violence and those reporting a single episode of violence within one year.

Contrary to previous findings that physical violence is more prevalent among women with low education,^29^,^30^ our results showed that women in The Gambia who had a secondary education are associated with recurrent injuries from physical violence. One possible explanation is that higher educational attainment of women is an indicator of autonomy and advocation for their rights, which may have resulted in resistance in African society where traditional sociocultural norms propagating gender inequality still exist, thus increasing the risk of recurrent violent injuries.^31^ However, women with tertiary education did not exhibit the same result. Another possible explanation for this finding is that less educated women triggered fewer violent confrontations because they tended to be less assertive and more willing to conform to the social expectation that women are to be submissive to their partners.

A polygamous family and living in an extended family compound in Africa partly reflect religious beliefs and traditional family structures in The Gambia. More than 90% of the Gambian population practice Islam and often practice polygamy. Polygamous families have a large family size, which has been strongly associated with violence against women.^32^,^33^,^34^ In a polygamous family or large extended-family compound, contentious rivalry among co-wives living under one roof is common due to taking turns in cooking, sharing facilities, gossiping, and teasing, as well as other issues related to quarrels between children, which could result in repeated physical confrontations among them or with their male spouse. In addition, because The Gambia is a patriarchal society, this result potentially relates to the importance of social norms around family kinship, such that issues of land use and inheritance by male children cause frequent physical violence among co-wives or with their spouse.

Single-parent families have a high risk of financial hardship and poor parental monitoring.^35^ Children who lack adequate parental supervision may be influenced by delinquent peers engaging in risky behaviors and thus increase the risk of recurrent violence in adult life.^36^ Furthermore, several studies have indicated that compared with women raised by two biological parents, those raised in single-parent families were more likely to have externalized and internalized disorders, lower social competence, and lifetime exposure to several forms of violence in adult life.^37^,^38^ Alternatively, women pampered by parents are more likely to be violent than their counterparts,^39^ and the pampering effect might be stronger in a single-parent family as compensation for the lost time and attention. Studies in the United States and Brazil have revealed that women residing in rented houses experienced five times the physical violence experienced by women living in owned houses.^40^,^41^ The effects of living in rented housing on recurrent violence partly reflects that low- and middle-income families seeking job opportunities but unable to own a house in the metropolitan areas in The Gambia are prone to intra-family violence. Rented houses in The Gambia are often overcrowded and poorly managed, and issues of shared common spaces and household utilities (eg, electricity and water bills) may cause physical confrontation among women. In addition, given that 53% of women in this study were married, the financial stress of meeting daily family needs and paying rent on time might result in displaced aggression and physical confrontations of men with their marital partners.

Similar to the results of studies that were done in the Middle East and South Asia,^42^,^43^ in this study most women with recurrent physical violence lived with their mothers-in-law (51.8%). Several studies have highlighted parents-in-law as instigators of conflict in the couple’s relationship and their role in recurrent violence against women.^12^,^44^ In The Gambia, violence against women might result from the controlling behaviors of mothers-in-law due to overprotectiveness and emotional attachment toward their sons. Furthermore, misunderstandings between daughters-in-law and parents-in-law, such as words or deeds misperceived as disrespect to parents, may have resulted in recurrent violence against women by their partners. Childhood abuse has been associated with numerous adverse adult-health outcomes and has been consistently identified as a strong predictor for physical violence against women.^45^,^46^ Women abused in childhood had an increased probability of internalizing and externalizing behavior problems and risk of incident and recurrent violence in adult life.^47^,^48^

## LIMITATIONS

This study has several limitations. First, although bed partition curtains were closed to prevent potential observation of the participants by unauthorized persons during the interviews and the controls were carefully interviewed to ensure they did not experience multiple violence, it is still possible that some NVC injuries might actually have resulted from violence and some VC injuries might have been recurrent but were not disclosed because the participants were concerned about confidentiality; thus, these potential misclassifications might have led to underestimation of the effects of risk factors identified for recurrent violent injuries. Second, recurrent injuries from physical violence might have been underreported by the two control groups partly because of the fear of negative reactions from the family after disclosing intrafamily violence caused by the spouse or other family members. Third, the generalizability of the results is somewhat limited because only female patients from public health facilities in urban and peri-urban areas were recruited, and those treated in private health facilities or those who live in rural areas might have different risk factors. Fourth, we did not measure alcohol use and substance abuse because the prevalences of the two behavioral characteristics are low in The Gambia, in contrast to high-income western countries where alcohol use and substance abuse are contributing factors to recurrent violent injuries.^6^–^8^ Finally, the characteristics of perpetrators were unmeasured in the study, and their education level, employment status, and alcohol use might have confounded the results.^49^

## CONCLUSION

Risk factors for recurrent violence may differ from those for a single episode of violence. African women in The Gambia may be at an increased risk of recurrent injuries if they belong to a polygamous family, have high educational levels, were raised by a single parent, live in a rented house, or live with in-laws. Intervention strategies targeting these factors could be effective for the prevention of violence against African women.

## Figures and Tables

**Table 1:** Comparisons of sociodemographic factors of the case group with the two control groups.

Characteristics	Cases (N = 116) n (%)	Violence controls (N = 116) n (%)	*P*-value	Nonviolence controls (N = 116) n (%)	*P*-value
Age (years)					
15–24	47 (40.5)	44 (37.9)	0.973	44 (37.9)	1.00
25–34	50 (43.1)	51 (44.0)		51 (44.0)	
35–44	16 (13.8)	18 (15.5)		17 (14.7)	
≥45	3 (2.6)	3 (2.6)		4 (3.5)	
Ethnicity					
Mandinka	36 (31.0)	32 (27.6)	0.090	56 (48.3)	0.06
Wolof	21 (18.1)	37 (31.9)		28 (24.1)	
Fula	24 (20.7)	16 (13.8)		13 (11.2)	
Others^a^	35 (30.2)	31 (26.7)		19 (16.4)	
Educational level					
Primary or no education	25 (21.6)	36 (31.0)	0.032	31 (26.7)	0.01
Secondary education	67 (57.8)	47 (40.5)		43 (37.1)	
Tertiary education	24 (20.7)	33 (28.5)		42 (36.2)	
Type of family origin					
Monogamous	57 (49.1)	91 (78.5)	0.001	86 (74.1)	<0.001
Polygamous	59 (50.9)	25 (21.5)		30 (25.9)	
Marital status					
Married	61 (52.6)	62 (53.4)	0.895	65 (56.0)	0.19
Single	55 (47.4)	54 (46.6)		51 (44.0)	
Age at first marriage					
<18 years	5 (8.2)	9 (14.5)	0.270	3 (5.9)	0.64
≥18 years	56 (91.8)	53 (85.5)		48 (94.1)	
Employment status					
Employed	54 (47.0)	50 (43.4)	0.419	42 (37.5)	0.64
Unemployed	13 (11.3)	3 (17.7)		6 (5.4)	
Home maker	16 (13.9)	30 (16.8)		28 (25.0)	
Student	32 (27.8)	24 (22.1)		36 (32.1)	
Household income^b^ (<GMD15,000)	87 (75.0)	96 (82.8)	0.148	92 (82.2)	0.14
Living in an extended family compound	33 (28.5)	13 (11.4)	0.001	10 (4.6)	<0.001
Number of male siblings (>2)	98 (84.5)	95 (81.9)	0.598	62 (53.5)	0.18
Number of female siblings (>2)	102 (87.9)	96 (82.8)	0.265	64 (55.2)	0.02
Raised in childhood					
Both parents	83 (71.6)	104 (89.7)	0.001	103 (93.6)	0.01
Single parent	20 (17.2)	5 (4.3)		5 (4.6)	
Relatives	13 (11.2)	7 (6.0)		2 (1.8)	
Body mass index (kg/m^2^), mean ± SD	23.2 ± 5.7	23.7 ± 6.0	0.522	23.9 ± 7.3	0.37

a Other ethnic groups include Jola, Serahuli, Manjago, Serer, Aku, and Balanta.

b The exchange rate was US dollar 1.0 = GMD 45.0.

*GMD*, Gambian dalasi; *kg*, kilogram; *m^2^*, height squared; *SD*, standard deviation.

**Table 2:** Comparisons of social characteristics of the case group with two control groups.

Characteristics	Cases (N= 116) n (%)	Violence controls (N=116) n (%)	*P* value	Nonviolence controls (N=116) n (%)	*P* value
Perceived social support, mean ± SD					
Family	22.8 ± 5.7	22.7 ± 5.6	0.88	24.2 ± 4.9	0.04
Friends	22.2 ± 5.4	22.8 ± 5.3	0.39	23.8 ± 4.6	0.09
Significant others	22.7 ± 5.6	22.6 ± 5.8	0.88	22.7 ± 5.3	0.09
Risk-taking behaviors, mean ± SD					
Social	13.6 ± 5.9	13.2 ± 6.3	0.61	13.2 ± 7.6	0.73
Recreational	9.28 ± 5.9	10.1 ± 6.9	0.31	10.8 ± 8.6	0.14
Health and safety	11.3 ± 6.2	11.6 ± 6.8	0.74	11.3 ± 6.2	0.95

*SD*, standard deviation.

**Table 3:** Comparisons of family factors of the case group with the control group, which consisted of women who had experiercned one episode of violent injury in the prior 12 months.

Characteristics	Cases (N=116) n (%)	Violence controls (N=116) n (%)	*P* value
Residential status			
Family house	38 (32.8)	56 (48.3)	<0.001
Own house	9 (7.8)	26 (22.4)	
Rented house	69 (59.5)	34 (14.7)	
Living with in-law in the past 12 months	64 (55.2)	35 (30.2)	<0.001
Witnessing parental violence	71 (61.2)	45 (38.8)	<0.001
Being physically abused as a child	63 (54.3)	28 (24.1)	<0.001

**Table 4:** Results of the conditional logistic regression analysis of risk factors with the adjusted odds ratio and 95% confidence interval for comparing the case group with the two control groups.

	Violence controls			Nonviolence controls		
		
Characteristics	OR	(95% CI)	*P* value	OR	(95% CI)	*P* value
Educational level						
Primary or no education	1.00	reference group		1.00	reference group	
Secondary education	6.47	(2.23–18.8)	<0.001	4.22	(2.67–10.7)	<0.001
Tertiary education	3.11	(0.96–10.1)	0.06	1.36	(0.51–3.67)	0.54
Type of family origin (polygamous/monogamous)	3.81	(1.42–10.3)	0.01	3.53	(1.56–8.00)	<0.001
Living in an extended family compound		N.A.		4.77	(2.73–13.2)	<0.001
Number of female siblings (>2/≤2)		N.A.		4.07	(2.81–9.17)	<0.001
Raised in childhood by						
Both parents	1.00	Reference group		1.00	Reference group	
Single parent	5.25	(2.08–18.9)	0.04	5.04	(2.43–17.8)	0.01
Family relative^a^	1.09	(0.21–3.80)	0.88	3.52	(1.00–12.4)	0.04
Residential status						
Family house	1.00	Reference group				
Own house	0.36	(0.09–1.37)	0.13		N.A.	
Rented house	4.74	(1.90–11.8)	<0.001		N.A.	
Living with in-law in the prior 12 months	5.98	(2.14–16.7)	<0.001		N.A.	
Being physically abused as a child	2.48	(1.01–6.10)	0.05		N.A.	

a Relatives include grandparents, aunts, and uncles.

*OR*, odds ratio; *CI*, confidence interval; *N.A.*, not available.

## References

[1] Brooke BS, Efron DT, Chang DC (2006). Patterns and outcomes among penetrating trauma recidivists: it only gets worse. J Trauma.

[2] Skogstad L, Tøien K, Hem E (2014). Psychological distress after physical injury: a one-year follow-up study of conscious hospitalised patients. Injury.

[3] World Health Organization (WHO) (2014). Global status report on violence prevention 2014.

[4] Faergemann C, Lauritsen JM, Brink O (2009). Demographic and socioeconomic risk factors of adult violent victimization from an accident and emergency department and forensic medicine perspective: a register-based case-control study. J Forensic Leg Med.

[5] Kimerling R, Alvarez J, Pavao J (2007). Epidemiology and consequences of women’s revictimization. Women’s Health Issues.

[6] Sonis J, Langer M (2008). Risk and protective factors for recurrent intimate partner violence in a cohort of low-income inner-city women. J Family Violence.

[7] Hankin A, Wei S, Foreman J (2014). Screening for violence risk factors identifies young adults at risk for return emergency department visit for injury. West J Emerg Med.

[8] Kaufman E, Rising K, Wiebe DJ (2016). Recurrent violent injury: magnitude, risk factors, and opportunities for intervention from a statewide analysis. Am J Emerg Med.

[9] Mears DP, Carlson MJ, Holden GW (2001). Reducing domestic violence revictimization: the effects of individual and contextual factors and type of legal intervention. J Interperson Violence.

[10] Boyle A, Frith C, Edgcumbe D (2010). What factors are associated with repeated domestic assault in patients attending an emergency department? A cohort study. Emerg Med J.

[11] Kuijpers KF, Van der Knaap LM, Winkel FW (2012). Victims’ influence on intimate partner violence revictimization: an empirical test of dynamic victim-related risk factors, an empirical test of dynamic victim-related risk factors. J Interperson Violence.

[12] Chan KL, Brownridge DA, Tiwari A (2008). Understanding violence against Chinese women in Hong Kong: an analysis of risk factors with a special emphasis on the role of in-law conflict. Violence Against Women.

[13] Desai S, Arias I, Thompson MP (2002). Childhood victimization and subsequent adult revictimization assessed in a nationally representative sample of women and men. Violence Vict.

[14] Flury M, Nyberg E, Riecher-Rössler A (2010). Domestic violence against women: definitions, epidemiology, risk factors and consequences. Swiss Med Wkly.

[15] Griffin RL, Davis GG, Levitan EB (2014). The effect of previous traumatic injury on homicide risk. J Forensic Sci.

[16] Krause ED, Kaltman S, Goodman LA (2008). Avoidant coping and PTSD symptoms related to domestic violence exposure: a longitudinal study. J Trauma Stress.

[17] Nygaard RM, Marek AP, Daly SR (2018). Violent trauma recidivism: Does all violence escalate?. Eur J Trauma Emerg Surg.

[18] Perez S, Johnson DM (2008). PTSD compromises battered women’s future safety. J Interpers Violence.

[19] Richards MH, Larson R, Miller BV (2004). Risky and protective contexts and exposure to violence in urban African American young adolescents. J Clin Child Adolesc Psychol.

[20] Adegoke TG, Oladeji D (2008). Community norms and cultural attitudes and beliefs factors influencing violence against women of reproductive age in Nigeria. Europ J Sci Res.

[21] Ilika AL (2005). Women’s perception of partner violence in a rural Igbo community. Afr J Reprod Health.

[22] Sardinha L, Nájera Catalán HE (2018). Attitudes towards domestic violence in 49 low- and middle-income countries: a gendered analysis of prevalence and country-level correlates. PLoS One.

[23] Gambia Bureau of Statistics (2013). The Gambia 2013 Population and Housing Census Preliminary Results.

[24] Nakigudde J, Musisi S, Ehnvall A (2009). Adaptation of the multidimensional scale of perceived social support in a Ugandan setting. Afr Health Sci.

[25] Stewart RC, Umar E, Tomenson B (2014). Validation of the multi-dimensional scale of perceived social support (MSPSS) and the relationship between social support, intimate partner violence and antenatal depression in Malawi. BMC Psychiatry.

[26] Weber EU, Blais A, Betz N (2002). A domain-specific risk-attitude scale: measuring risk perceptions and risk behaviors. J Behav Decis Mak.

[27] Szrek H, Chao LW, Ramlagan S (2012). Predicting (un)healthy behavior: a comparison of risk-taking propensity measures. Judgm Decis Mak.

[28] Maldonado G, Greenland S (1993). Simulation study of confounder-selection strategies. Am J Epidemiol.

[29] Fageeh WM (2014). Factors associated with domestic violence: a cross-sectional survey among women in Jeddah, Saudi Arabia. BMJ Open.

[30] Tadesse BT, Dachew BA, Bifftu BB (2015). High incidence of interpersonal violence in Northwest Ethiopia: a cross-sectional study. Int Emerg Nurs.

[31] Kalaca S, Dundar P (2010). Violence against women: the perspective of academic women. BMC Public Health.

[32] Ali AA, Yassin K, Omer R (2014). Domestic violence against women in Eastern Sudan. BMC Public Health.

[33] Koenig MA, Ahmed S, Hossain MB (2003). Women’s status and domestic violence in rural Bangladesh: individual- and community-level effects. Demography.

[34] Rao V (1997). Wife-beating in rural south India: a qualitative and econometric analysis. Soc Sci Med.

[35] Stack RJ, Meredith A (2018). The impact of financial hardship on single parents: an exploration of the journey from social distress to seeking help. J Fam Econ Issues.

[36] Singh A, Kiran UV (2014). Effect of single parent family on child delinquency. Inter J Sci Res.

[37] Bae H, Solomon PL, Gelles RJ (2009). Multiple child maltreatment recurrence relative to single recurrence and no recurrence. Child Youth Serv Rev.

[38] Finkelhor D, Ormrod RK, Turner HA (2007). Poly-victimization: a neglected component in child victimization. Child Abuse Negl.

[39] Mueller D (2011). Pampered children and the impact of parenting styles.

[40] Lacey KK, West CM, Matusko N (2016). Prevalence and factors associated with severe physical intimate partner violence among U.S. Black women: a comparison of African American and Caribbean Blacks. Violence Against Women.

[41] Vieira EM, Perdona Gda S, Santos MA (2011). Factors associated with intimate partner physical violence among health service users. Rev Saude Publica.

[42] Gangoli G, Rew M (2011). Mothers-in-law against daughters-in-law: domestic violence and legal discourses around mother-in-law violence against daughters-in-law in India. Wom Stud Inter Forum.

[43] Haj-Yahia MM (2000). Wife abuse and battering in the sociocultural context of Arab society. Fam Process.

[44] Clark CJ, Silverman JG, Shahrouri M (2010). The role of the extended family in women’s risk of intimate partner violence in Jordan. Soc Sci Med.

[45] Springer KW, Sheridan J, Kuo D (2003). The long-term health outcomes of childhood abuse. An overview and a call to action. J Gen Intern Med.

[46] Bensley L, Van Eenwyk J, Wynkoop Simmons K (2003). Childhood family violence history and women’s risk for intimate partner violence and poor health. Am J Prev Med.

[47] Vézina J, Hébert M (2007). Risk factors for victimization in romantic relationships of young women: a review of empirical studies and implications for prevention. Trauma Violence Abuse.

[48] Widom CS, Czaja SJ, Dutton MA (2008). Childhood victimization and lifetime revictimization. Child Abuse Negl.

[49] Kyriacou DN, Anglin D, Taliaferro E (1999). Risk factors for injury to women from domestic violence. N Engl J Med.

